# Integrated Bioinformatics Analysis and Target Drug Prediction of Inflammatory Bowel Disease Co-existent Diabetes Mellitus

**DOI:** 10.2174/0115734099282247231211111219

**Published:** 2024-01-03

**Authors:** Lili Yang, Ning Wang, Yutong Wang, Wen Li, Ziyang Kong, Bin Zhang, Yaoyao Bian

**Affiliations:** 1 Jingwen Library, Nanjing University of Chinese Medicine, Nanjing, 210023, China;; 2 Jiangsu Provincial Engineering Center of TCM External Medication Researching and Industrializing, Nanjing University of Chinese Medicine, Nanjing, 210023, China;; 3 School of Acupuncture-Moxibustion and Tuina, School of Health Preservation and Rehabilitation, Nanjing University of Chinese Medicine, Nanjing, 210023, China;; 4 Department of Gastroenterology, Ningbo Municipal Hospital of TCM, Affiliated Hospital of Zhejiang Chinese Medical University, Ningbo, 315012, China

**Keywords:** Inflammatory bowel disease, diabetes mellitus, integrated bioinformatics analysis, targeted drug prediction, differentially expressed genes, gene expression omnibus public database

## Abstract

**Introduction:**

Inflammatory bowel disease (IBD) has become one of the public problems worldwide and its incidence rate is increasing year by year. Its concomitant disease *i.e*. diabetes mellitus (DM) has attracted more and more attention due to DM altering the progression of IBD and leading to long periods of intermittent recurrence and deterioration. The common mechanism and potential target drug of IBD with comorbid chronic conditions of DM were explored.

**Methods:**

Gene expression profile data were downloaded from the Gene Expression Omnibus (GEO) public database. The differentially expressed genes (DEGs) were identified by R software. GO annotation and pathway enrichment were performed, a protein-protein interaction (PPI) network was constructed, associated lncRNAs were predicted and drug prediction targeting key genes was made. Additionally, the regulatory network among core genes, associated pathways, and predicted lncRNA in IBD with coexistent DM were visualized.

**Results:**

We identified the critical gene MMP3 with lncRNA CDKN2BAS involved in the PPAR pathway, which uncovered the underlying regulatory mechanism of IBD with coexistent DM. We also predicted the potential therapeutic compound ZINC05905909 acting on MMP3.

**Conclusion:**

Our findings revealed the regulatory mechanism chain of critical gene MMP3, lncRNA CDKN2BAS, and PPAR pathway and provided potential therapeutic compound ZINC05905909 for drug therapy to treat comorbid IBD DM.

## 1 INTRODUCTION

Inflammatory bowel disease (IBD) including ulcerative colitis (UC) and Crohn's disease (CD) is a kind of chronic and non-specific intestinal inflammation disease with alternating onset and remission of intestinal inflammation [1]. The symptoms of IBD range from mild to severe, including abdominal pain, diarrhea, weight loss, fever, and gastrointestinal bleeding. So far, the most commonly acceptable hypothesis about the pathophysiology of IBD is that environmental risks on genetically vulnerable individuals lead to epithelial barrier deficiencies and microbiota disorders, and ultimately result in dysregulated immune responses. It is estimated that 1.6 million Americans are diagnosed with IBD each year [2]. IBD has become one of the public problems worldwide and its incidence rate is increasing year by year. Its concomitant disease such as diabetes mellitus (DM) has attracted more and more attention due to DM altering the progression of IBD and leading to long periods of intermittent recurrence and deterioration [3].

DM is one of the systemic inflammatory disorders characterized by hyperglycemia which is caused by inefficient insulin secretion or insulin resistance. The prevalence of DM rose rapidly worldwide from 415 million in 2015 to 642 million in 2040 reported by the International Diabetes Federation [4]. It is estimated that 17.7 million Americans were diagnosed with DM in 2000 and this number is expected to be 30.3 ~ 36 million by 2030 [5]. In China, 30% elderly population (more than 60 years old) are diabetic according to the report of the seventh national census, and more than 50% of diabetic patients were newly diagnosed [6].

A recent cohort study indicated that IBD patients with coexistent DM had increased use of healthcare and reduced quality of life [7], and a higher risk of cardiovascular disease and sepsis complications than the IBD-only cohort [8]. Inflammatory biomarkers such as C-reactive protein, erythrocyte sedimentation rate, eosinophilia, and monocytosis altered significantly in the patients with IBD DM when compared with the IBD patients only, evidenced by another longitudinal cohort study [9]. Moreover, a recent study suggested that current IBD medications had potential effects on regulating glycemic metabolism [10]. Increasing evidence showed that there could be some co-existence between DM and IBD because both of them are affected by genes, gut microbiota, and lifestyle [7, 11]. However, the common mechanism of IBD with comorbid chronic conditions of DM is limited. In this study, we downloaded gene expression profile data of IBD patients from the Gene Expression Omnibus (GEO) public database and identified differentially expressed genes (DEGs) with integrative bioinformatics methods. In addition, potential regulatory pathways, core genes as biomarkers, related lncRNA, and targeted drug predictions of core genes were predicted. The close relationship among key genes, related lncRNAs, and enriched pathways in IBD DM were verified by text mining in an exploratory approach. We tried to elucidate the potential regulatory mechanistic connection between IBD and DM.

## 2 MATERIALS AND METHODS

### 2.1 Microarray Data Acquisition

The microarray expression profile datasets (GSE96665 [12], GSE59071 [13], GSE53306 [14]) were downloaded from the GEO public database (available online: https://www.ncbi.nlm.nih.gov/gds). The above three microarray data came from human tissues. The platform for the GSE96665 dataset was based on Agilent-028004 SurePrint G3 Human GE 8x60K Microarray (Feature Number version), which consisted of 12 active samples, 8 inactive samples, and 15 controls. The platform for the GSE59071 dataset was based on (HuGene-1_0-st) Affymetrix Human Gene 1.0 ST Array (transcript (gene) version), which contained 82 active samples, 23 inactive samples, and 11 controls. The platform for the GSE53306 dataset was based on the Illumina HumanHT-12 WG-DASL V4.0 R2 expression bead chip, which consisted of 16 active samples, 12 inactive samples, and 12 controls.

### 2.2 Data Processing and DEGs Screening

The downloaded raw data were background corrected, normalized, standardized, and logarithmically converted. The matrix files downloaded from the platform were converted by using the R software and annotation package. The probe names corresponding to ID were converted to international standard gene names (gene symbols) and saved in the TXT files. DEGs were screened by using the limma package (available online: http://www.bioconductor.org/) from the Bioconductor software package. *p*-value < 0.05 and log fold change (FC) > 2 were set as the screening threshold. In addition, volcano maps of the differential genes were drawn by using the ggplot 2 package.

### 2.3 Integration of Microarray Data

DEGs in the three microarray datasets analyzed by limma packages were separately saved as TXT files. The RRA software package was used to analyze the three sets of DEGs. A list of up-regulated and down-regulated DEGs in the three chips was used for the following analyses.

### 2.4 GO Ontology (GO) Functional Annotation and KEGG Pathway Enrichment Analysis

GO annotation was used to identify the descriptions of genes that had three biological processes, *i.e*. biological process (BP), cellular component (CC), and molecular function (MF). The functions of candidate genes were annotated by the cluster profile package [15]. KEGG pathway enrichment was performed to present functional annotation and classifications, which was performed by the ggplot2 package [16]. The altered genes were significantly up-regulated and down-regulated from integrated microarray data. *p*-value < 0.05 was considered as the cutoff criterion.

### 2.5 LncRNA Prediction

Screened DEGs were imported into the STARBASE database (available online: http://starbase.Sysu.edu.cn/) to predict the associated lncRNA. To improve the accuracy of the prediction results, the lncRNAs validated in at least one or more diseases were selected.

### 2.6 Protein-protein Interaction (PPI) Network Integration

STRING database (available online: http://string.embl.de/) was a comprehensive database to identify and visualize the interactions between known proteins and predicted proteins [17]. The integrated results in this database came from experimental data, predicted bioinformatic data, and other databases. The proteins corresponding to the central node may be core proteins or key candidate genes with important physiologically regulatory functions. The core of the Cytoscape software is the network, where each node is a gene, protein, or molecule and the connections between the nodes represent the interactions between these biomolecules. Cytoscape software (available online: http://www.cytoscape.org) was used to construct the interaction relationships of core genes and predicted lncRNA.

### 2.7 Targeted Drugs Prediction

500 small molecules from the ZINC Database (available online: http://zinc.docking.org/) [18] were selected and imported into ChemBio3D Ultra 14.0 for energy minimization. The Minimum RMS Gradient was set to 0.001 and the small molecules were saved in mol2 format. The optimized small molecules were imported into AutodockTools-1.5.6 for hydrogenation, charge calculation, charge allocation, and saved in ‘pdbqt’ format. The MMP3 target protein (PDB ID: 4G9L) was downloaded from the PDB database (available online: http://www.rcsb.org/). Protein crystal water and raw ligands were removed by using Pymol2.3.0. The protein structure was imported into AutoDocktools (v1.5.6) for hydrogenation, charge calculation and charge allocation, and saved in ‘pdbqt’ format. AutoDock Vina1.1.2 was used for molecular docking. The relevant parameters of MMP3 targets were set as center_x = 22.531, center_y = 69.212, center_z = 104.252, and the lattice box was set to 50×50×50 (The spacing of each lattice point was 0.375Å) and the remaining parameters were default. The highest-scored conformations were imported into PyMOL2.3.0 and LigplotV2.2.4 for interaction pattern analysis.

## 3 RESULTS

### 3.1 Identification of Altered Expressed Genes

The microarray datasets GSE96665, GSE59071, and GSE53306 were background-corrected, standardized, and log-transformed, as shown in Fig. (**1**). One thousand seven hundred and sixty-seven altered expressed genes were identified in the GSE96665 dataset, including 950 up-regulated genes and 817 down-regulated genes. One thousand two hundred and thirty-eight altered expressed genes were screened from the GSE59071 dataset, including 793 up-regulated genes and 445 down-regulated genes. Moreover, 953 altered expressed genes were identified in the GSE53306 dataset, including 495 up-regulated genes and 458 down-regulated genes. The heat maps of the altered expressed genes in the three microarrays were presented in Fig. (**2**).

### 3.2 Integration of DEGs by Integrative Bioinformatics

Three chip datasets were analyzed by using the Limma package, sorted by log fold-change values, and then integrated and analyzed with the RRA method. The RRA method was based on the assumption that each gene in each experiment was randomly ordered. If a gene ranked high in all experiments, the smaller the *p*-value, the greater the likelihood of differential gene expression. By using the RRA analysis, we identified 33 DEGs, which contained 24 up-regulated genes (including DUOXA2, DUOX2, SLC6A14, TNIP3, NOS2, REG3A, LCN2, PI3, ABCA12, REG1A, MMP3, TCN1, GNA15, MMP7, COL1A1, C4BPB, DEFA5, REG1B, CLDN2, MMP1) and 9 down-regulated genes (AQP8, UGT2A3, PCK1, ABCG2, TMIGD1, SLC26A2, CLDN8, ADH1C, SLC37A2). Heatmap was plotted with R-heatmap software (top 20), as shown in Fig. (**3**).

### 3.3 GO Functional Annotation and KEGG Enrichment Analysis

The biological annotation of the above DEGs screened from integrated bioinformatic methods was conducted. The results of GO functional annotation showed these DEGs were enriched in the 12 biological processes, 7 molecular functions, and 7 cell components. Among the GO terms, collagen catabolic process (MMP7, COL1A1, MMP3, MMP, MMP1) and extracellular matrix disassembly (MMP7, MMP3, MMP1) were highly significantly annotated, as shown in Fig. (**4**). The distribution of DEGs in the different GO functional terms was shown in Fig. (**5**). The above DEGs were markedly enriched in 18 significant signaling pathways, including ABC transporter (ABCG2, ABCA12) and peroxisome proliferator-activated receptor (PPAR) signaling pathway (MMP3, PCK1), as presented in Fig. (**6**).

### 3.4 PPI Network Construction and Key Gene Screening

To annotate protein cellular localization and biological function, the PPI network was constructed after removing the isolated and partially connected nodes by using the STRING online tool, as shown in Fig. (**7**). There were 53 differentially expressed genes (nodes) and 111 interaction relationships (edges) in the PPI network. Among them, COL1A1 (degree=9), MMP1 (degree=8), MMP3 (degree=8), and MMP7 (degree=8) were at the core of the network and were closely associated with other genes. Additionally, we found that MMP3 was one of the DEGs in the integrated bioinformatic results, and it was also involved in the GO biological annotation of collagen catabolic process and extracellular matrix disassembly, and KEGG enrichment of the PPAR signaling pathway. Herein, we inferred that MMP3 might play an important role in the occurrence and development of IBD, which might serve as a potential therapeutic target.

### 3.5 LncRNA Prediction and mRNA-lncRNA Network Construction

Five eligible lncRNAs (TMED10P, NCRNA00092, DIO3OS, CYP2B7P1, CDKN2BAS) were predicted by the STARBASE online database. The target genes (mRNAs) and the predicted lncRNA were constructed by using Cytoscape software. The mRNA-lncRNA network was presented in Fig. (**8**). We found that three lncRNAs (DIO3OS, CDKN2BAS, and CYP2B7P1) were closely related to MMP3.

### 3.6 Targeted Drug Prediction

In this study, 500 small molecules were screened based on the ZINC database, and the compounds with the higher scores were obtained. The top 10 compounds were ZINC05905909, ZINC05093720, ZINC06076810, ZINC06111515, ZINC05904760, ZINC06013503, ZINC05 903864, ZINC05918221, ZINC06076808, ZINC06142470. Among them, ZINC05905909 got the highest score and its structure is shown in Fig. (**9A**). The interaction pattern was analyzed by using PyMOL2.3.0 and LigplotV2.2.4. It revealed that the binding energy of ZINC5905909 and MMP3 was -11.4kcal/mol, which suggested a good binding effect. ZINC5905909 interacted with MMP3 mainly through the formation of hydrogen bonds and hydrophobic force. The binding cavity was formed by His201 (B), His224 (B), Thr227 (B), Glu216 (B), Ala217 (B), Leu218 (B), Tyr220 (B), Leu222 (B), Glu202 (B), Tyr223 (B), Leu164 (B), Val198 (B), Leu197 (B), Asp228 (B), Phe232 (B), and Arg233 (B). Eight hydrogen bonds were formed with His201 (B), His224 (B), Thr227 (B), Glu216 (B), Ala217 (B), Leu218 (B), and Tyr220 (B). The lengths of the hydrogen bond were 3.29 Å, 3.20 Å, 3.14 Å, 3.68 Å, 3.20 Å, 3.21 Å, 3.61 Å, and 3.46 Å, respectively. There were also some hydrophobic effects with Leu222 (B), Glu202 (B), Tyr223 (B), Leu164 (B), Val198 (B), Leu197 (B), Asp228 (B), Phe232 (B), and Arg233 (B), as shown in Fig. (**9B**). The predicted results of the above molecular docking suggested that ZINC05905909 might be a potential drug targeting MMP3.

### 3.7 Texting Mining

Text mining was used to explore the close connections among key genes, related lncRNAs, and enriched pathways in IBD DM by using Coremine Medical (available online: http://www.coremine.com/medical/) [19]. Co-occurrence analysis of the literature was conducted using inflammatory bowel disease, diabetes mellitus, core gene (MMP3), five predicted lncRNAs (TMED10P, NCRNA00092, DIO3OS, CYP2B7P1, CDKN2BAS), and the significantly enriched PPAR pathway as search terms. We found that MMP3, lncRNA (CDKN2BAS), and the PPAR pathway were closely related to IBD DM, which formed a ‘pentagram’, as presented in Fig. (**10**). Consequently, all the data showed that MMP3, CDKN2BAS, and the PPAR pathway might display a regulatory mechanism in the pathological mechanism of IBD with coexistent DM.

## 4 DISCUSSION

In the current study, we found a potential regulatory mechanism of IBD with coexistent DM in an exploratory approach. The integrated analysis of differential gene expression profiles is a good method to identify reliable molecular markers of diseases [20]. As far as we know, this is the first study to uncover the underlying mechanism of comorbid IBD DM at the molecular level by using integrated informatics methods. We analyzed different expression patterns between the active and inactive samples in three datasets (GSE96665, GSE59071, GSE53306) by using the RRA method. The RRA method is used to integrate microarray data on the basis of the *p*-value of a gene ranked in the altered expressed genes, which suggests the correlation of the gene associated with IBD. The smaller the *p*-value, the higher the ranking, and the more likelihood of the gene with IBD. After integration, a total of 33 altered expressed genes were screened. Significantly, MMP3 was identified as a candidate gene among the differentially expressed genes in the pathogenesis of IBD by integrated bioinformatics analysis. We found that MMP3 ranked 11^th^ in up-regulated DEGs. GO functional annotation showed that MMP3 was enriched in the biological process of the collagen catabolic process. The results of KEGG pathway enrichment suggested that the altered expressed genes were markedly enriched in the PPAR signaling pathway, and MMP3 was annotated in this PPAR pathway. In addition, we found that MMP3 was the core gene in the PPI network. Collectively, all evidence indicated that MMP3 could serve as a key biomarker in the pathologic process of IBD. Long non-coding RNAs (lncRNAs) are the RNA fragments of non-coding proteins longer than 200 nucleotides. Mounting evidence indicates that lncRNA plays an important role in the occurrence and development of IBD [21, 22]. We used target genes to predict lncRNA and got five eligible lncRNAs including TMED10P, NCRNA00092, DIO3OS, CYP2B7P1, and CDKN2BAS. Among these predicted lncRNAs, DIO3OS, CDKN2BAS, and CYP2B7P1 were closely related to MMP3. Interestingly, co-occurrence analysis of text mining results further gave us a clear visualization of the correlated network of the core gene, predicted lncRNA, and associated pathway in IBD co-existent DM.

Our current findings indicated that MMP3 might be a crucial gene in IBD co-existent DM in an initial approach view. MMP3 is one of the enzymes of Matrix Metallopeptidases (MMPs), which is the main enzyme group of extracellular matrix (ECM) and basement membrane components. It is involved in tissue remodeling and plays an important role in the occurrence and development of inflammatory diseases [23]. MMP3 was involved in the submucosal repair process of the ulcer basement of IBD. It is detected in the lamina propria below the basement membrane in the area of mucosal injury [24]. Moreover, MMP3 was overexpressed in the tissues and serum of IBD patients, suggesting that MMP3 involved the ECM remodeling process of the inflammatory colon mucosa in IBD patients, and could serve as an important marker [25-27]. Coincidentally, MMP3 was supported to be an independent indicator of prognosis in asymptomatic DM [28]. Genetic polymorphism studies showed that the 6A allele of MMP3 was associated with the increased risks of arterial calcification in DM patients in Japan [29], and was an independent risk factor for coronary artery stenosis in DM patients in Irian [30]. However, no data has been studied on the underlying molecular mechanism of MMP3 in IBD patients with DM. Further correlation analysis between MMP3 and IBD severity is needed to elucidate the role of MMP3 in comorbid IBD DM.

Of note, lncRNA CDKN2BAS was predicted to be closely related to MMP3. CDKN2B-AS1 was found significantly downregulated in the colon tissues of active IBD cases by transcriptional studies [31, 32]. Interestingly, CDKN2B-AS1 was also identified with increased susceptibility to type 2 DM, evidenced by a genome-wide association study [33]. Additionally, we found that the PPAR signaling pathway also played a significant role in the regulatory mechanism of IBD co-existent DM. The critical mechanism of lncRNA CDKN2BAS interacting with MMP3 needs to be explored in future experimental studies. PPAR pathway is one of the well-documented pathways either in metabolic disorders such as DM or chronic inflammatory diseases such as IBD [34]. It suggested that PPAR indeed played an important role in managing glucose and controlling colonic inflammation. However, no study has been found on studying the critical effects of PPAR on the comorbid IBD DM. Our study highlighted important regulatory connections among MMP3, CDKN2B-AS1, and PPAR in IBD co-existent DM, which might provide a new research direction for future studies to elucidate the molecular mechanism of IBD DM comorbidity.

Currently, gene identification by high-throughput screening methods and the design of drugs targeting these genes might be novel treatments for disease therapy [35]. Molecular docking is a very important approach in drug virtual screening. To identify potential therapeutic drugs as far as possible, potential compounds from the ZINC database were screened. We found that ZINC05905909 could serve as a potential molecular targeting MMP3 based on the virtual screening of molecular docking compounds. It can be hypothesized that the identification of ZINC05905909 might have high potential effects on MMP3 activity and could be further explored for the therapeutic management of IBD DM. The above findings need to be validated by further experiments. However, our study comes with several limitations. Firstly, we didn’t provide experimental or computational evidence to support our initial findings. Future studies either *in vitro* or deeper *in silico* analyses are needed. Secondly, as far as we know, molecular dynamics simulations play key roles in evaluating the molecular flexibility of ligands and receptors. The conformational changes will influence the results of the docking study. Herein, a theoretical approach to select conformations from the docking simulations is very important to determine the theoretical accuracy [36-38]. Our study didn’t use molecular dynamics simulations to predict the target drug. Further studies with selecting confirmation from docking simulations are needed.

## CONCLUSION

**Fig. (11):**
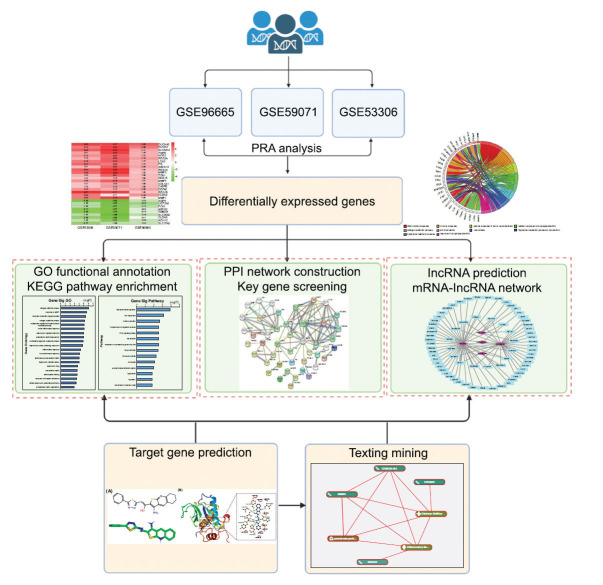
The workflow of integrated bioinformatics analysis and target drug prediction for IBD co-existent DM.

In conclusion, we identified the critical gene MMP3 with predicted lncRNA CDKN2BAS involved in the PPAR pathway that uncovered the common regulatory mechanism of IBD with coexistent DM. We also predicted the potential therapeutic compound ZINC05905909 acting on MMP3 (Fig. **11**). Our findings might provide new insights into elucidating the underlying mechanism of IBD co-existent DM for future mechanistic studies, and possible novel strategies for drug therapy to treat comorbid IBD DM. Future experimental and preclinical studies are warranted to confirm the critical effects of these novel targets.

## Figures and Tables

**Fig. (1):**
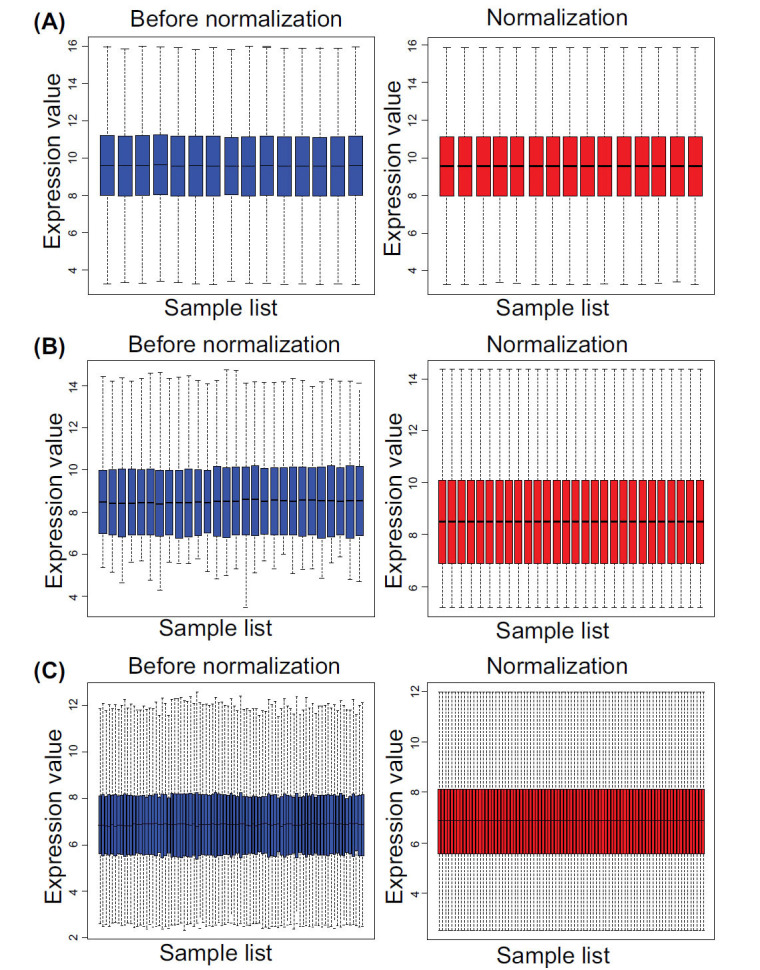
Standardization of gene expression. (**A**) The standardization of the microarray dataset GSE96665. (**B**) The standardization of the microarray dataset GSE59071. (**C**) The standardization of the microarray dataset GSE53306. The blue box plots displayed the data before normalization, while the red box plots displayed the data after normalization.

**Fig. (2):**
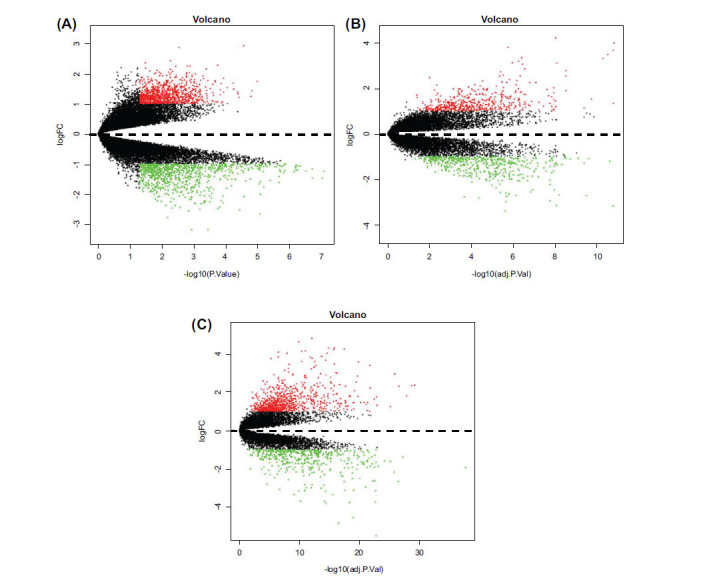
Volcano map of the altered expressed genes. (**A**) Volcano plot of the altered expressed genes in microarray dataset GSE96665. (**B**) Volcano plot of the altered expressed genes in microarray dataset GSE59071. (**C**) Volcano plot of the altered expressed genes in microarray dataset GSE53306. The red points displayed the up-regulated genes, the green points displayed the down-regulated genes, and the black points displayed genes with no significant difference.

**Fig. (3):**
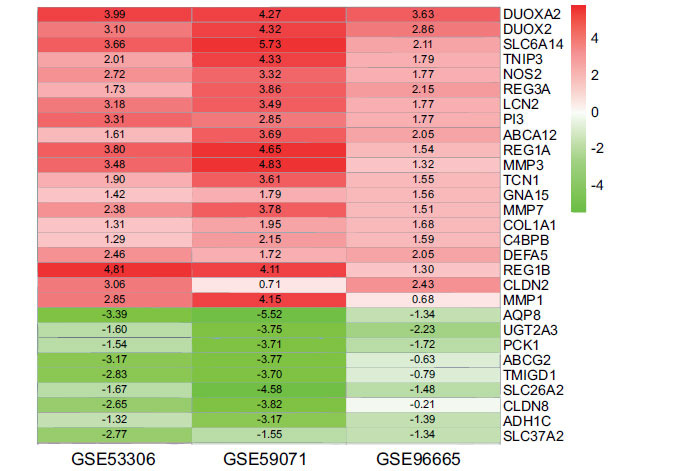
LogFC heatmap of the three expression microarray data. The abscissa represented the GEO ID. The ordinate represented the gene name. Red displayed logFC > 0 and green displayed logFC < 0. The values in the box represented the logFC values.

**Fig. (4):**
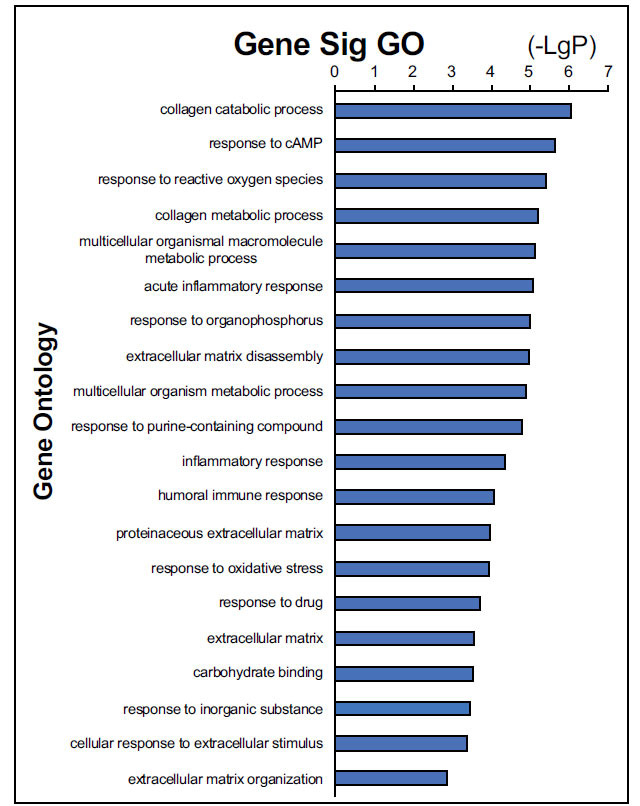
GO functional annotation of differentially expressed genes (top 20).

**Fig. (5):**
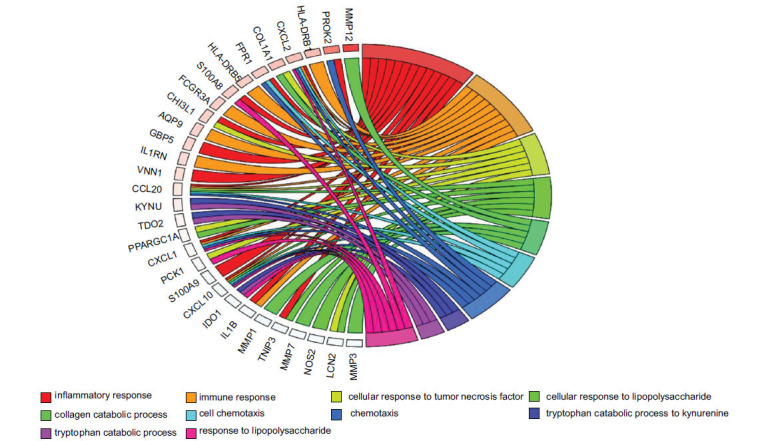
Distribution of the differentially expressed genes in the different GO functional annotations.

**Fig. (6):**
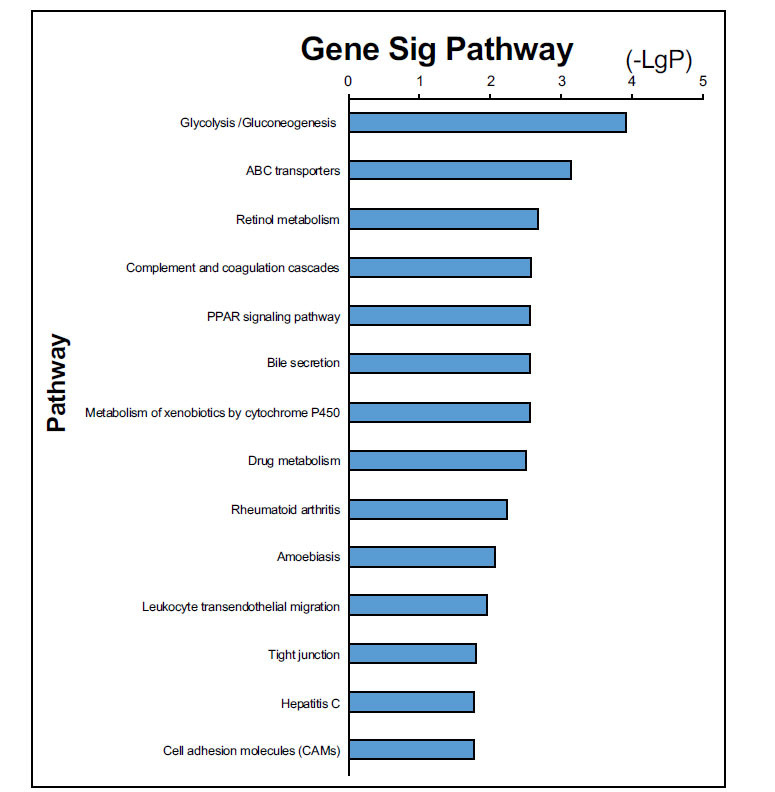
KEGG pathway enrichment of differentially expressed genes.

**Fig. (7):**
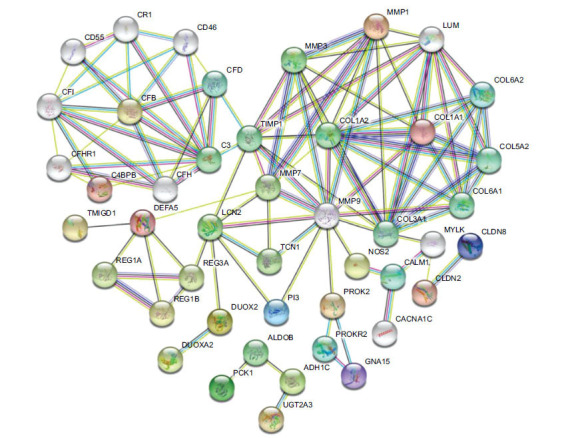
Differentially expressed PPI network by STRING online tool. Fifty-three differentially expressed genes (nodes) and 111 interaction relationships (edges) were constructed.

**Fig. (8):**
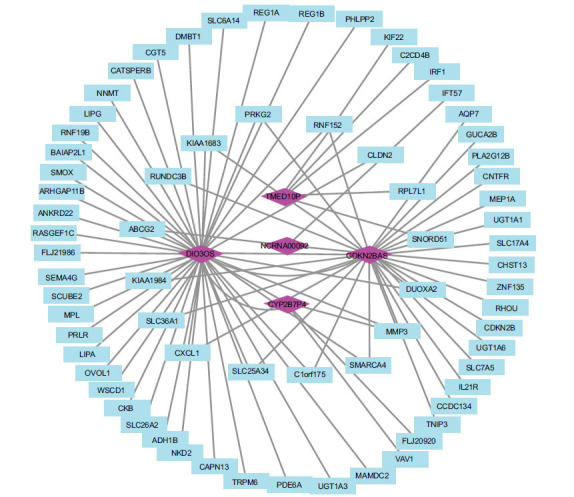
mRNA-lncRNA network constructed by Cytoscape software. The purple diamond represented lncRNAs and the blue rectangle represented mRNA.

**Fig. (9):**
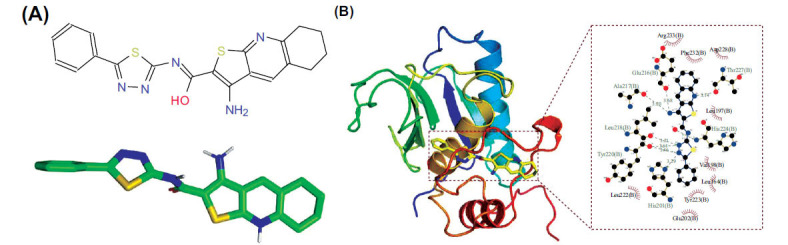
Potential predicted compounds and the interaction pattern of the predicted compound with MMP3 by PyMOL2.3.0 and LigplotV2.2.4. (**A**) The structure of predicted compound ZINC5905909. (**B**) The hydrophobic effects of ZINC5905909 with MMP3.

**Fig. (10):**
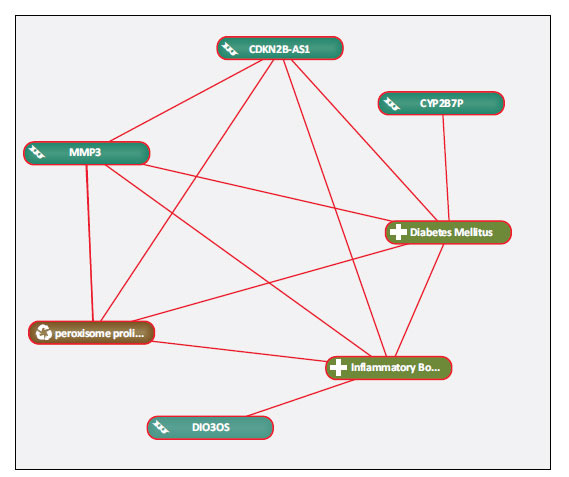
Linear association among core genes, predicted lncRNAs, associated pathway in IBD with coexistent DM using Coremine medical online tool.

## Data Availability

The data and supportive information are available within the article.

## LIST OF ABBREVIATIONS

CD: Crohn's Disease
DEGs: Differentially Expressed Genes
DM: Diabetes Mellitus
ECM: Extracellular Matrix
FC: Fold Change
IBD: Inflammatory Bowel Disease
lncRNAs: Long Non-coding RNAs
MMPs: Matrix Metallopeptidases
PPAR: Peroxisome Proliferator-activated Receptor
PPI: Protein-protein Interaction
UC: Ulcerative Colitis
